# Curcumin-loaded nanostructured systems for treatment of leishmaniasis: a review

**DOI:** 10.3762/bjnano.15.4

**Published:** 2024-01-04

**Authors:** Douglas Dourado, Thayse Silva Medeiros, Éverton do Nascimento Alencar, Edijane Matos Sales, Fábio Rocha Formiga

**Affiliations:** 1 Department of Immunology, Aggeu Magalhães Institute (IAM), Oswaldo Cruz Foundation (FIOCRUZ), 50670-420 Recife, PE, Brazil; 2 Department of Pharmacy, Federal University of Rio Grande do Norte (UFRN), 59010180, Natal, RN, Brazil; 3 College of Pharmaceutical Sciences, Food and Nutrition. Federal University of Mato Grosso do Sul (UFMS), 79070-900, Campo Grande, MS, Brazil; 4 University Ruy Barbosa (UniRuy), 41720-200, Salvador, BA, Brazil; 5 Faculty of Medical Sciences (FCM), University of Pernambuco (UPE), 50100-130, Recife, PE, Brazil

**Keywords:** antiparasitic, *Curcuma longa*, curcuminoids, leishmaniasis, nanocarriers, neglected tropical diseases

## Abstract

Leishmaniasis is a neglected tropical disease that has affected more than 350 million people worldwide and can manifest itself in three different forms: cutaneous, mucocutaneous, or visceral. Furthermore, the current treatment options have drawbacks which compromise efficacy and patient compliance. To face this global health concern, new alternatives for the treatment of leishmaniasis have been explored. Curcumin, a polyphenol obtained from the rhizome of turmeric, exhibits leishmanicidal activity against different species of *Leishmania* spp*.* Although its mechanism of action has not yet been fully elucidated, its leishmanicidal potential may be associated with its antioxidant and anti-inflammatory properties. However, it has limitations that compromise its clinical use. Conversely, nanotechnology has been used as a tool for solving biopharmaceutical challenges associated with drugs, such as curcumin. From a drug delivery standpoint, nanocarriers (1–1000 nm) can improve stability, increase solubility, promote intracellular delivery, and increase biological activity. Thus, this review offers a deep look into curcumin-loaded nanocarriers intended for the treatment of leishmaniasis.

## Introduction

Neglected tropical diseases (NTDs) comprise a group of 20 diseases that are caused, in most cases, by viruses, fungi, bacteria, or parasites, such as helminths and protozoa. They affect mainly women and children from impoverished communities. Leishmaniasis is a NTD that has affected more than 350 million people worldwide, with worrisome 700,000 to one million new cases annually [1–2].

This tropical disease is caused by a vector-borne protozoan parasite of the genus *Leishmania* which is transmitted by the bite of female sandflies. Different species of *Leishmania* spp*.* can cause specific clinical manifestations. These are (i) cutaneous leishmaniasis, which can be the localized type when the lesions are limited to certain areas of the skin, or diffuse when several lesions occur over an extensive area of skin tissue [3]; (ii) mucocutaneous leishmaniasis, which causes total or partial degeneration of the mucous membranes of the nose, mouth, and throat [4], and (iii) visceral leishmaniasis (also known as kala-azar), can cause systemic infection affecting the liver, spleen, hematogenous and lymphatic systems [5–6].

For the treatment of these infections, therapies based on pentavalent antimony (first-line drug treatment), amphotericin B, miltefosine, and paromomycin have been employed [7]. Despite being effective, these drugs cause cardiotoxicity, renal, pancreatic, and liver toxicity, and teratogenicity. Furthermore, cases of drug resistance are already well reported for antileishmanial drugs, such as the pentavalent antimonial salts [8].

Therefore, finding new therapeutic alternatives for this neglected tropical disease continues to be of utmost importance [9]. Current studies have highlighted curcumin as a promising antiparasitic alternative [10]. Curcumin (diferuloylmethane) is a yellow polyphenol extracted from the rhizome of *Curcuma longa*, popularly known as turmeric [11–13]. This molecule presents a high tolerance profile, and it has shown *in vitro* and *in vivo* leishmanicidal properties against different species of *Leishmania* spp*.* [11,14].

Despite its pharmacological potential, curcumin has some physicochemical and biopharmaceutical limitations that should be highlighted, such as: (i) low aqueous solubility, (ii) poor gastrointestinal absorption, (iii) high rates of metabolism, (iv) inactivity of metabolic products, and (v) rapid elimination and clearance [15–16].

To get around these several limitations, nanotechnological systems such as nanoemulsions [17], microemulsions [18], self-nanoemulsifying systems [19], nanoparticles [20], nanoliposomes [21], micelles [22], and nanocrystals [23] have been utilized. These systems can promote (i) protection of the drug against degradation in physiological media, (ii) increase in drug solubility, and (iii) modification/targeting of the drug enabling transport through biological membranes [13,24].

Therefore, this review focuses on mapping the nanotechnologies used to load curcumin and discussing the increase in the leishmanicidal properties of this drug according to its nanostructured vehicles.

## Review

### Leishmaniasis: general aspects

Leishmaniasis is a neglected tropical disease caused by a flagellated protozoa of the genus *Leishmania*. The genus belongs to the Trypanosomatidae family, and it is transmitted by insect vectors of the genus Phlebotomus (in the Old World) or Lutzomyia (in the New World) [25]. The disease is present in several countries and it has affected more than 350 million people worldwide. Its incidence has increased more than 40-fold in the last 20 years, making it the second most prevalent parasitic disease in the world after malaria [2,26–27].

The disease can manifest in three different forms: cutaneous leishmaniasis (CL), which is the most abundant form; mucocutaneous leishmaniasis (MCL); and visceral leishmaniasis (VL), which is the severe and lethal form of the disease, with a mortality rate above 95% [28]. The form in which the disease manifests itself in the patient is determined mainly by immunological aspects and general health conditions of the host and by the species of the parasite. In general, CL and MCL are caused by *L. tropica*, *L. major*, *L. amazonensis,* and *L. brasiliensis*. Meanwhile, VL is caused by *L. donovani*, *L. infantum,* and *L. chagasi*. However, there are reports of cutaneous leishmaniasis caused by the *L. donovani* and *L. infantum* complex. This is due to advances in the molecular detection of these species worldwide [29–31].

The *Leishmania* spp*.* life cycle is mainly divided into two evolutionary stages: (i) extracellular promastigote, which is the flagellate form and is in the intestine of the invertebrate host; (ii) intracellular amastigote, a spherical form that is found in cells of the vertebrate host. Infected sandflies inject blood with the parasite in promastigote form into the vertebrate host, which causes macrophages or other cells of the mononuclear phagocytic system to phagocytose the promastigotes. The *leishmania* spp. cells then differentiate into amastigotes inside the phagocytic cells, multiply by binary fission until the host cell breaks down and releases the parasites to infect other cells and tissues [2,32–33].

The Food Drug Administration (FDA) recommends five drugs for the treatment of leishmaniasis: pentavalent antimonials, amphotericin B, pentamidine, paromomycin, and miltefosine. Among these drugs, only the pentavalent antimonials were designed for leishmaniasis, while the other four were initially approved to treat other diseases. Although used in different treatment protocols, most are only capable of controlling the infection and relieving symptoms, while displaying concerning toxicity and numerous therapeutic limitations [34–36].

Furthermore, treatment abandonment and failure due to drug resistance are two of the problems encountered with the usual treatments. Thus, seeking therapeutic alternatives to those currently available, many natural or synthetic molecules have been studied and evaluated for their antileishmanial potential, among which curcumin may be featured [4].

### Curcumin and its antileishmanial properties

**Figure 1:**
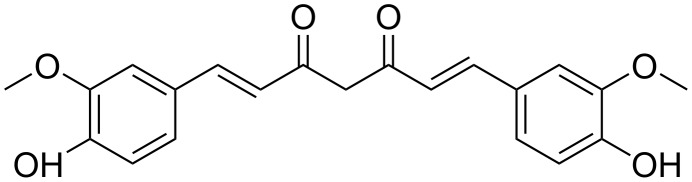
Chemical structure of curcumin.

Curcumin (curc) is a polyphenol (Figure 1) obtained from the rhizome of *Curcuma longa* and is the main curcuminoid present in this plant [11–12]. Due to its good tolerance profile and safety even at high doses (12 g/day), curc has been extensively studied as a therapeutic agent [11,14]. Numerous preclinical and clinical trials have concluded that curc has great potential for the treatment of various diseases in humans [37–39].

Among the diverse biological potential of this molecule, its antiparasitic properties against different diseases have attracted considerable attention in recent decades [10,40–41]. Among such, the potential of curc against cutaneous and visceral leishmaniasis has been explored [42]. *In vitro* and *in vivo* studies have revealed that curc displays leishmanicidal activity against amastigotes and promastigotes of the species *Leishmania amazonensis* [43]*, Leishmania braziliensis* [44], *Leishmania donavani* [45], *Leishmania infantum* [46], *Leishmania major* [46–47], and *Leishmania tropica* [46,48].

**Figure 2:**
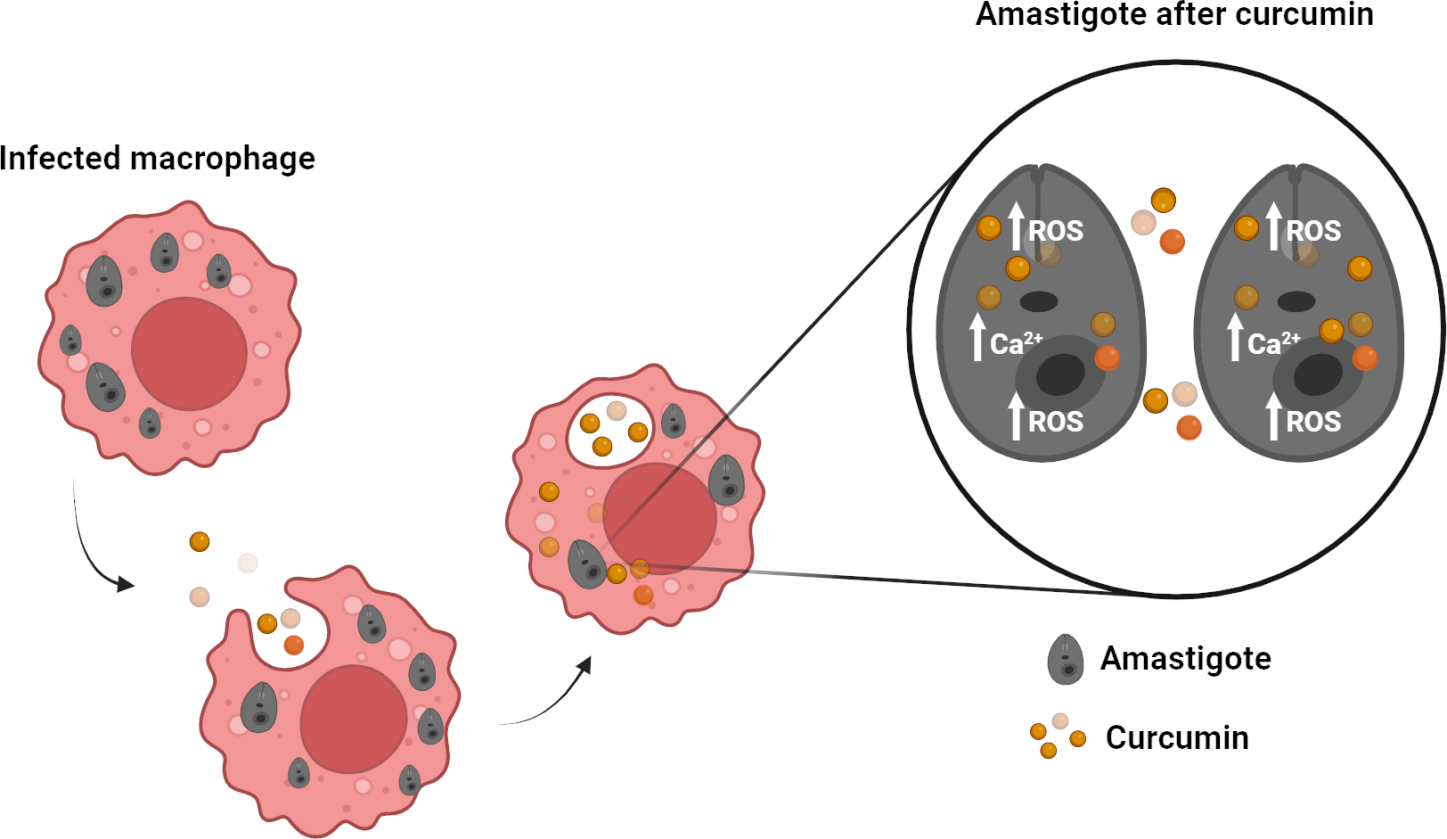
Possible mechanisms of action of curcumin against leishmaniasis. Created with BioRender.com (https://biorender.com/). This content is not subject to CC BY 4.0.

Although proposed mechanisms of action are not fully elucidated, curc has been shown to have antileishmanial effects through its anti-inflammatory and antioxidant properties [42,47,49–50]. Figure 2 reveals the possible mechanisms of action of this drug against leishmaniasis.

Curc inhibits the activation of nuclear factor-κB (NFκB); the production of TNFα, IFNγ, and nitric oxide; and gene expression of inducible nitric oxide synthase (iNOS) [49,51]. These pro-inflammatory factors are related to the parasitic infection of leishmaniasis. Additionally, this molecule produces reactive oxygen species (ROS) and elevates cytosolic calcium. These occur in the exposure of phosphatidylserine to the outer plasma membrane leaflet and DNA fragmentation, causing the death of the leishmaniasis parasite [47,52].

Despite its promising antileishmanial potential, curc has several drawbacks, such as: (i) low aqueous solubility, (ii) rapid clearance, (iii) low tissue absorption, and (iv) notable chemical degradation (neutral and alkaline pH), which severely reduces its bioavailability and hinder its clinical use [18,53–54]. Given this scenario, approaches such as carrying curc in nanostructures have been used to overcome such drawbacks.

### Nanostructured systems for the treatment of leishmaniasis

The existing treatments for leishmaniasis (cutaneous, mucocutaneous, or visceral) are still insufficient or frequently ineffective. This is due to several limitations of the drugs used, such as (i) high levels of toxicity and prolonged treatment time, which leads patients to discontinue treatment, (ii) high cost of treatment and parasite resistance to drugs, which is a major issue and occurs as a result of genetic mutations that reduce the response of the parasite towards a given drug through decreased uptake of the drug by macrophages [55–57].

Thus, nanotechnology-based systems are a promising alternative for drug delivery and vectorization in the treatment of leishmaniasis as they present several advantages. One could mention decreased side effects, modified drug release, prevention of rapid metabolization, protection of photosensitive molecules, the ability to deliver multiple antileishmanial drugs that can have a synergistic effect, and increased solubility, which results in increased bioavailability. These advantages are determined by the physicochemical properties of the systems, and by the release and targeting features of the loaded nanosystems, rather than by the drug properties themselves [58–62].

The intracellular amastigote form of *Leishmania* spp*.* allocates in macrophage phagolysosomes from infected individuals [63]. However, the intracellular uptake of bioactive molecules is especially hindered for hydrophobic molecules [64], making it difficult for the drug to reach the parasite. On the other hand, nanocarriers can target the interior of macrophages residing in the spleen, liver, and bone marrow, effectively delivering antileishmanial drugs to such sites. Overall, drug targeting results in increased treatment efficacy and reduced toxicity, mostly by reducing drug doses and preventing its interaction with unwanted receptors [30,65].

In this scenario, active targeting happens by the functionalization of nanocarriers, making drug delivery specific to macrophage targets, such as ᴅ-mannose, phosphatidylserine, or lactoferrin. This may reduce the drug resistance of the parasite in the long term. Furthermore, the surface charge of nanostructures may influence internalization since positive charges favor electrostatic interactions of these carriers with the macrophage membrane. As a result, the macrophages uptake the drug-loaded nanocarrier by phagocytosis, where they will directly act on the parasites [65–67].

Several types of nanosystems have been studied for carrying antileishmanial drugs, such as polymeric nanoparticles, lipid nanoparticles, nano- and microemulsions, liposomes, or metallic nanoparticles [68]. Costa-Lima and colleagues incorporated bisnaphthalimidopropyldiaaminooctane (BNIPDaoct) into PLGA polymeric nanoparticles and obtained particles with sizes around 150 nm, with encapsulation efficiency around 90% [69]. BNIPDaoct is a bisnaphthalimidopropyl derivative, which acts on the life cycle of *Leishmania infantum*. Therefore, the authors evaluated the antileishmanial potential of the formulations and the free molecule in amastigote forms of *Leishmania infantum*. The study showed an eight- to ten-fold decrease in macrophage cytotoxicity of the nanoencapsulated molecule when compared to its free form. In addition, the uptake of these nanoparticles by macrophages was higher than that by fibroblasts, with an IC_50_ approximately two times lower than that of the free drug, and a selectivity index 20 times higher. *In vivo* studies demonstrated that the nanoformulations were more effective in reducing parasitemia in the spleen, with results equivalent to the group treated with amphotericin B.

Das and collaborators, on the other hand, addressed the development of nanostructured lipid carriers (NLC) with ursolic acid (UA) functionalized with *N*-octyl chitosan. The NLC were approximately 143 nm of the hydrodynamic diameter. The authors also found encapsulation and drug-load efficiencies of 88.63 ± 2.70% and 12.05 ± 0.54%, respectively [70]. They evaluated the antileishmanial potential of the nanosystems against resistant strains of *Leishmania donovani*, which resulted in a 15-fold improvement in drug activity when into NLC, with a selectivity index for the intracellular model in macrophages almost three times higher than that of the free drug. *In vivo* studies showed that the suppression of parasite load in the spleen of mice was around 60% for free-UA and close to 90% for NLC-UA. Confocal microscopy images proved the cell uptake of NLC into macrophages.

Metal nanoparticles are also excellent alternatives for carrying antileishmanial drugs [71]. Almayouf et al. produced silver nanoparticles (Ag-NP) with sizes ≈100 nm by green synthesis using extracts of *Ficus carica* Linn and *Olea europaea* L., which are rich in phenolic compounds. The authors also evaluated the antileishmanial potential of the nanosystems regarding concurrent treatment or pretreatment against *Leishmania major* cutaneous infection in female Balb/c mice [72]. The results of the study showed an improvement in skin lesions of groups treated with Ag-NP both before and after infection. The group treated after infection displayed a significant decrease in lesion size starting in the second week of treatment, with complete healing after 21 days, while the group treated with Pentostan healed after 28 days.

Moraes and colleagues prepared nanoemulsions (NE) of andiroba oil (*Carapa guianensis* Aublet, anoandi) and copaiba oil (*Copaifera sp*. Linnaeus, nanocopa) and tested their effects against *L. infantum* and *L. amazonensis* promastigotes and intracellular amastigotes, as well as the effects of oral administration of the formulations in infected mice [73]. The droplet size of the NEs was 76 and 88 nm for nanocopa and nanoandi, respectively. The authors observed a significant decrease in parasite load for both investigated species when treated by both nanoandi and nanocopa. Moreover, there was a decrease in lesion size and parasite load from the liver and spleen of mice treated with NE. In ultrastructural analysis performed by scanning electron microscopy, it was possible to observe morphological changes, oval aspect, and disappearance of the flagellum in the promastigote parasites treated with doses of NE above the IC_50_.

Peixoto and collaborators developed epoxy-α-lapachone-loaded microemulsions (ME). They assessed the ME *in vivo* performance against *L. amazonensis* in infected BALB/c mice. The ME droplet size was smaller than 120.4 ± 7.7 nm and displayed a good stability profile over 73 days. The *in vivo* studies demonstrated that after two weeks of treatment, BALB/c mice infected with *L. amazonensis* showed a decrease in paw lesions (about two-fold) in response to microemulsion, compared to the untreated group. Additionally, the amount of parasites in the lymph nodes (31.5%) and footpad (60.3%) decreased [57].

Another nanostructured platform explored for the delivery of leishmanicidal drugs is the liposomal platform [74]. Artemisinin-loaded nanoliposomes smaller than 100 nm were obtained and evaluated in a murine model infected with *L. donovani*. Artemisinin-NPs reduced the number of *ex vivo* infected macrophages and the intracellular infection of *Leishmania donovani* amastigotes (IC_50_ of 6.0 ± 1.4 µg/mL and 5.1 ± 0.9 µg/mL, respectively). Artemisinin-NPs showed better efficacy than free artemisinin after therapy in a mouse model of visceral leishmaniasis. Percentages of inhibition in the liver and spleen were 82.4 ± 3.8% and 77.6 ± 5.5%, respectively [75].

The results of the studies highlighted in this topic confirm the relevance of the use of nanostructured formulations for the delivery of bioactive compounds and uptake by macrophages, promoting an increase in antileishmanial efficacy, a decrease of toxicity, and, as a result, an increase in the selectivity index of these compounds. The same rationale is applied to the incorporation of curc in nanostructured systems, which will be discussed in the following topics.

### Nanostructured systems with curcumin for the treatment of leishmaniasis

**Figure 3:**
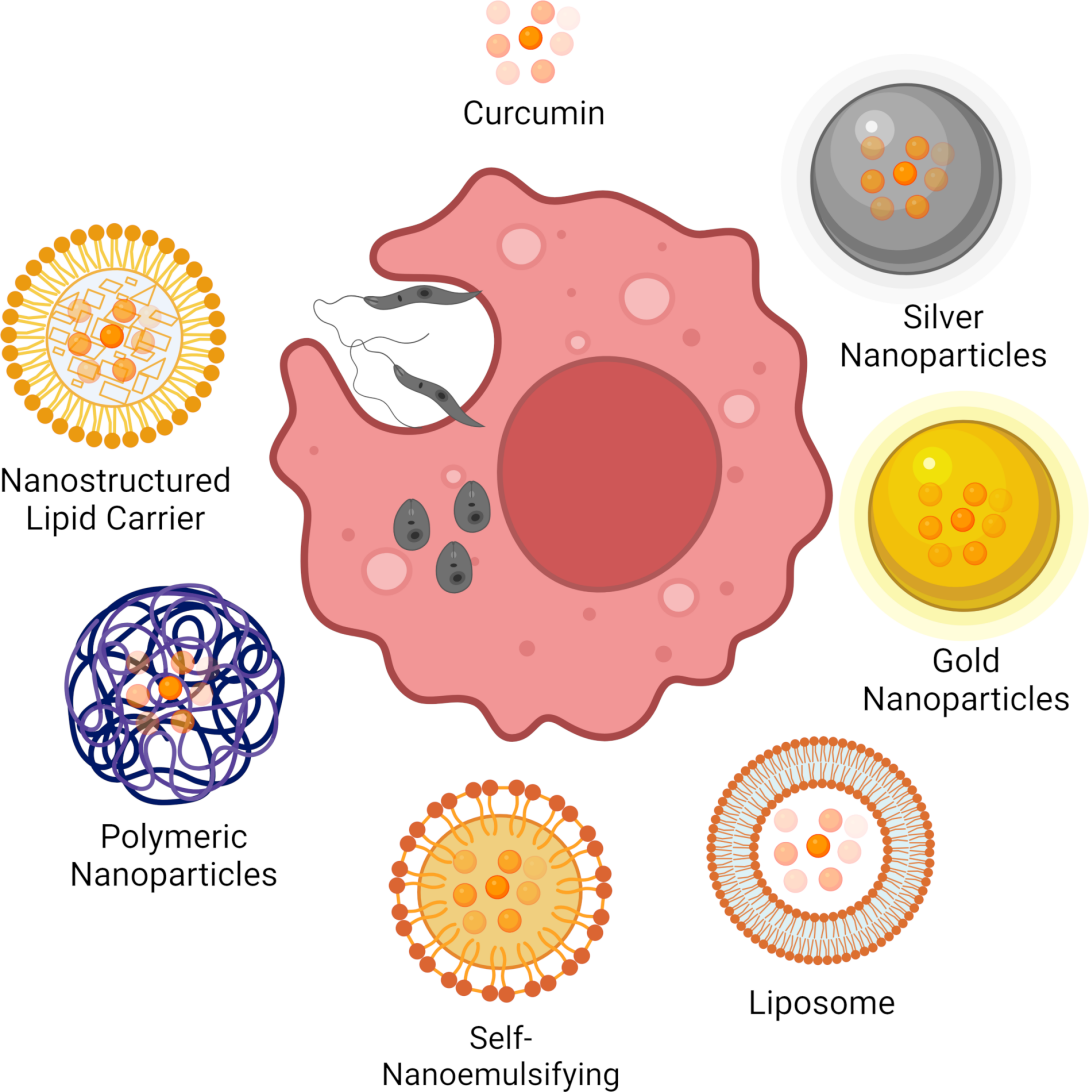
Overall scheme demonstrating curcumin-loaded nanostructured systems that have been assayed in vitro and/or in vivo against different *Leishmania* species for the treatment of leishmaniasis. Created with BioRender.com (https://biorender.com/). This content is not subject to CC BY 4.0.

Different nanostructured systems with curc intended for the treatment of leishmaniasis have been developed (Figure 3). The articles included in this work approach the following nanosystems: (i) self-nanoemulsifying drug-delivery systems (SNEDDSs), (ii) nanoliposomes, (iii) nanostructured lipid carriers, (iv) polymeric, and (v) metallic nanoparticles. Different performances of nanostructured systems containing curc are discussed in the section below.

### Self-nanoemulsifying drug-delivery systems

The SNEDDSs are lipid-based drug-delivery systems made of an isotropic blend of oils, surfactants, and co-surfactants or co-solvents [76]. These spontaneously form O/W nanoemulsions (≤200 nm) in aqueous media (generally in a physiological media) [77–78]. The SNEDDSs have been successfully used to carry molecules with biopharmaceutical limitations, improving their physicochemical and leishmanicidal properties [79–80].

In this context, curcumin-loaded SNEDDSs intended for the treatment of leishmaniasis were developed. Khan and collaborators developed curcumin-loaded self-emulsifying drug delivery systems (curc-SNEDDSs) for topical administration in cutaneous and mucocutaneous leishmaniasis [81]. The systems were produced by homogenization using a vortex mixer. The authors obtained two nanoformulations, which showed droplet size between 26–29 nm, polydispersity index (PdI) lower than 0.2, and zeta potential between −3.6 and −4.4 mV. The curc-SNEDDSs (1% of curc) promoted an increase in antileishmanial (*in vitro*) activity (IC_50_: 0.13–0.18 µg/mL and 0.25–0.27 µg/mL) when compared to curc in its free form (IC_50_: 22.50–24.60 µg/mL) against promastigote and amastigote forms of *Leishmania tropica*, respectively. The nanoformulations were able to kill the intracellular amastigotes in macrophages. Moreover, the authors developed a further study wherein seven nanoformulations with different mixtures of oils, surfactants, and co-surfactants were reported [48]. The obtained SNEDDSs showed droplet size between 30–80 nm and IC_50_ values of 0.26–0.36 μg/mL and 0.23–0.37 μg/mL against promastigotes and amastigotes of *Leishmania tropica*, respectively. However, in this study, they did not perform intracellular studies on infected macrophages. Nevertheless, both studies containing curcumin-loaded SNEDDSs promoted an increase in leishmanicidal activity.

Differently, Khan and collaborators also developed SNEDDSs for the entrapment of amphotericin B against *Leishmania tropica*. The authors also observed low IC_50_ values promoted by the nanosystems (two different SNEDDSs) against amastigote (IC_50_ values for SNEDDSs A and B, 0.025 and 0.056 µg/mL, respectively) and promastigote forms (IC_50_ values for SNEDDSs A and B, 0.017 and 0.031, respectively). These results evidenced the effectiveness of different SNEDDSs when compared to that of the control AmB-liposome AmBisome, which demonstrated IC_50_ values 10 times higher for amastigotes and promastigotes [79]. Although this nanostructured system is a promising carrier, further *in vitro* and *in vivo* studies are necessary to evaluate such improvements for curc or other leishmanicidal drugs.

### Nanoliposomes

Nanoliposomes are nanoscale lipid bilayer vesicles mainly composed of phospholipids. These systems (small liposomes: 20–100 nm and large liposomes: >100 nm) are capable of loading both hydrophilic and lipophilic drugs [82–83]. Leishmanial drugs, such as miltefosine [84], buparvaquone [85], nitazoxanide [86], artemisinin [75], berberine [87], and paromomycin [88] have already been successfully loaded into nanoliposomes promoting increased antiparasitic activity.

Recently, Bafghi and collaborators produced nanoliposomes for curc delivery as a new alternative for leishmaniasis treatment [89]. Briefly, the nanoliposomes were prepared by thin-film hydration, resulting in spherical vesicles with 176.5 nm of diameter, PdI <0.2, zeta potential of + 35 mV, and an entrapment efficiency of 92%. Curcumin-entrapped nanoliposomes showed leishmanicidal activity (*in vitro*) against the promastigotes of *Leishmania major*, whose IC_50_ values were 6.41, 3.80, and 2.33 μg/mL for the incubation times of 24, 48, and 72 h, respectively. Additionally, this nanosystem proved to be biocompatible with skin fibroblasts (*in vitro*). However, neither the cell viability of this system in healthy macrophages nor models of parasite infection in this cell type were evaluated. Considering that the nanoscale platform approved by the FDA and currently used in the treatment of leishmaniasis is liposomal amphotericin-B (IC_50_: 0.18 μg/mL) [90], this liposomal formulation containing curc should receive greater attention. In addition, more complex efficacy and safety studies (*in vivo*) must be conducted so that they could be transferred to the market following the Ambisome^®^ path.

### Nanostructured lipid carriers

Nanostructured lipid carriers (NLCs) are lipid-based formulations with a solid matrix at room temperature that differ from solid lipid nanoparticles when it comes to their matrix organizational level. Nanostructured lipid carriers offer advantages such as enhanced stability, low toxicity, increased shelf life, improved drug loading capacity, and biocompatibility over other conventional lipid-based nanocarriers, such as nanoemulsions and solid lipid nanoparticles [91]. Due to their properties, the use of NLCs has been a successful strategy for entrapping drugs with leishmanicidal activity [92–95]. Accordingly, Riaz and collaborators entrapped curc into NLCs to evaluate their activity in the treatment of cutaneous leishmaniasis (*L. tropica*) [96]*.* The authors obtained the NLCs by hot homogenization, resulting in structures with a hydrodynamic mean particle size of 312 ± 1.89 nm, PdI of 0.305 ± 0.17 and zeta potential of −38 ± 0.93 mV. These particles were able to entrap 88% of curc due to the irregular lipid crystal structure of NLCs [53]. The NLCs proved to be safe for macrophages, which promptly internalized the nanostructures, as proven by the strong intracellular fluorescence levels. Furthermore, NLCs increased the *in vitro* leishmanicidal activity of curc (IC_50_: 105.49 ± 3.71 μg/mL) when compared to curc-free NLCs (IC_50_: 165.06 ± 4.64 μg/mL). Similar performance occurred *in vivo* in axenic amastigotes, (IC_50_ of the curc-NLC: 190.3 ± 3.19 and IC_50_ value of the curc-free: 243.56 ± 2.89 μg/mL) corroborating the improvements in the leishmanicidal response after entrapment of curc in the nanostructure.

This biological performance was also observed in artemether-NLC against *L.infantum*. Nanostructured lipid carriers conferred an increase in the leishmanicidal activity of the drug. Artemether-NLCs presented IC_50_ values of *L. infantum* promastigote and amastigotes of 16.43 and 15.42 μg/mL, respectively. On the other hand, free artemether IC_50_ values were 2-fold higher (i.e., 37.12 and 32.10 μg/Ml). Finally, the nanostructured system revealed the lowest cytotoxicity against J774 macrophages [97].

Amphotericin B-loaded NLCs were also developed for the treatment of leishmaniasis. Free amphotericin B (AmB) and AmB-NLCs (250 nm) were evaluated for their leishmanicidal performance against the amastigote form and host cells. Unlike curc-NLCs, which revealed an increase in activity, AmB-NLCs obtained IC_50_ with efficacy equivalent to AmB-free against the amastigote form of *L. braziliensis* (11.7 ± 1.73 and 5.3 ± 0.55 ng/mL respectively). However, NLCs greatly increased drug selectivity (1046 versus 813), as macrophages revealed smaller toxicity indicators, without evidence of nitric oxide or TNFα production [95].

In this perspective, NLCs have been a nanoscale platform with promising results aimed at encapsulating anti-leishmanial drugs. This encourages further studies to better understand how NLCs might affect the mechanisms of action of curc when delivered via these nanostructured systems.

### Polymeric nanoparticles

Polymeric nanoparticles (PNPs) are colloidal systems made of natural or synthetic polymers [98]. These systems can encapsulate or adsorb active pharmaceutical ingredients (APIs) and macromolecules [99–101]. In addition, PNPs can impact specific drug release kinetics and increase biocompatibility [60]. Different biodegradable polymers have been used for the development of targeted PNPs for the treatment of leishmaniasis [102].

In this scenario, chitosan is an interesting polymer for NP synthesis due to its positive charge, which favors adsorption by negatively charged cell membranes [103]. Additionally, studies have revealed antileishmanial properties of this polymer against Leishmania parasites, making it attractive for the synthesis of NPs intended for this treatment [56,104].

Therefore, Chaubey and colleagues developed mannose-conjugated curcumin–chitosan nanoparticles (curc-MCNPs) intended for visceral leishmaniasis [50]. The selected nanoparticles (curc-MCNPs) presented spherical morphology, hydrodynamic mean particle size of 215 nm, PdI of 0.381, zeta potential of +24.37 mV, and entrapment efficiency of 82.12%. Curc-MCNPs showed a more effective uptake and pronounced *in vitro* leishmanicidal activity (curc-MCNPs, median effective dose (ED_50_): 0.518 ± 0.01 µg/mL) against *L. donovani* amastigotes than curc-chitosan nanoparticles (curc-CNPs, ED_50_: 1.87 ± 0.075 µg/mL) and free curc (ED_50_: 2.8 ± 0.03 µg/mL). Furthermore, the *in vivo* uptake study indicated that the endocytosis of the NPs effectively occurred within macrophages of the reticuloendothelial system. Moreover, in another work of the research group, the authors evaluated the *in vitro* and *in vivo* leishmanicidal efficacy and toxicity of these nanoparticles [105]. *In vivo* antileishmanial activity in hamsters demonstrated significantly greater suppression of parasite replication in the spleen with Cur-MCNPs compared to that of Cur-CNPs. In addition, no cytotoxic profile was observed *in vitro* in macrophages (J774A.1), which was also confirmed by the minimal cytotoxicity observed in *in vivo* studies.

Hence, chitosan-based nanoparticles are a good strategy for drug delivery intended to treat leishmaniasis. This polymer can stimulate macrophages to produce pro-inflammatory cytokines, as they bind to receptors present in the cells of the immune system [106]. In this way, there is an internalization of the NPs containing this polymer and delivery of the drug for leishmanicidal activity. Additionally, the conjugation of the nanoparticles with sugars, such as mannose, makes drug delivery targeted and specific to the macrophage receptor, increasing the uptake by the system and consequently the intracellular leishmanicidal activity [50,105].

Poly(lactic-co-glycolic) acid (PLGA) is another polymer used for the development of nanoparticles for the treatment of leishmaniasis [107–108]. PLGA is an FDA-approved polymer that is commonly used in the synthesis of nanoparticles due to its special features such as biocompatibility, biodegradability, low toxicity, and adjuvanticity [109–110]. Given these properties, Tiwari and co-workers produced curcumin-loaded Eudragit-PLGA-nanoparticles (curc-E-PLGA-NPs) and evaluated their leishmanicidal activity with miltefosine combination therapy. The authors functionalized the surface of PLGA-NPs with Eudragit L30D, a polymer that provides pH-dependent drug release and significantly improved targeted action, thus increasing the efficacy of the drug [45].

Curc-E-PLGA-NPs showed spherical morphology, with a hydrodynamic mean diameter of 182.3 ± 7.4 nm, PdI of 0.281 ± 0.015, zeta potential of −12.7 ± 0.141 mV, and entrapment efficiency of 93.2 ± 3.9 %. Curc-E-PLGA-NPs exhibited IC_50_ values *in vitro* of 1.34 ± 0.045 and 1.61 ± 0.032 µg/mL for promastigotes and amastigotes of *L. donovani*, respectively. Furthermore, the association of curc-E-PLGA-NPs with miltefosine revealed synergism in both promastigotes and amastigotes. In the *in vivo* hamster model, curc-E-PLGA-NPs also showed leishmanicidal activity, individually or associated with miltefosine. The synergy evidenced between these drugs (i) increased the production of toxic reactive oxygen/nitrogen metabolites, (ii) increased phagocytic activity, and (iii) increased lymphocyte proliferation [45]. Furthermore, curc-E-PLGA-NPs proved to be effective as an adjuvant in the therapy against leishmaniasis.

Like curc, other leishmanicidal drugs have been encapsulated into polymeric nanoparticles and shown promising results. Ghosh and collaborators encapsulated amphotericin B in PLGA-NPs by modifying its surface with mannose. As with curc nanoparticles functionalized with mannose, an increase in leishmanicidal activity was observed. AmB-free, AmB-PLGA-NPs, and mannose-PLGA-NPs presented IC_50_ values of 0.15 ± 0.08, 0.09 ± 0.07, and 0.07 ± 0.04 μM and selectivity index (SI) of 80, 255, and 314, respectively. In addition to the influence of the nanostructure, sugar promotes greater internalization of these nanoparticles due to the mannose receptors on the surface of macrophages [111].

Pentamidine-loaded nanoparticles also showed great results due to the nanostructure. Free pentamidine and Pentamidine-NPs were administered orally in an *in vivo* experimental model of mice infected with visceral leishmaniasis. Only the nanoencapsulated drug showed a significant reduction in the relative weight of organs such as the spleen and liver, commonly increased in visceral leishmaniasis [112]. Thus, polymeric nanoparticles have proven themselves as suitable for increasing the antileishmanial potential of compounds known to be used against *Leishmania*. Further, they are versatile and safe nanostructured systems that promote increased leishmanicidal activity and can encapsulate curc as a therapeutic alternative for the treatment of leishmaniasis.

### Metallic nanoparticles

Metallic nanoparticles (MNPs) are versatile nanostructures due to their tunability in shape, composition, size, structure, assembly, and optical properties [113]. These nanoformulations can be synthesized through chemical, physical, or biological processes and are solely generated from metal precursors such as silver and gold [114–115]. In addition, MNPs have been widely used in therapy, drug delivery, targeting, and imaging [116–117]. Current studies have directed the use of metallic nanoparticles such as silver and gold nanoparticles against *Leishmania* sp. [118]. As silver and gold nanoparticles can produce reactive oxygen species (ROS) and Leishmania is known to be extremely sensitive to these compounds, these have been promising nanoformulations in the treatment of leishmaniasis [118–121].

Given these properties, the synergistic activity of MNPs and leishmanicidal drugs has been evaluated, highlighting an increase in antileishmanial efficacy [122–123]. Accordingly, Badirzadeh and collaborators synthesized curc-coated silver nanoparticles (curc-AgNPs) for the treatment of cutaneous leishmaniasis [124]. Curc-AgNPs presented a spherical shape, 32 nm of diameter, and a zeta potential of −19.8 mV. This nanoformulation prevented the *in vitro* growth of *L. major* promastigotes and inhibited their viability (IC_50_: 58.99 μg/mL). In addition, it eliminated amastigotes inside macrophages (EC_50_: 57.14 μg/mL), which remained viable above 50% at concentrations below 307.16 μg/mL. Further, the authors carried out *in vivo* assays with BALB/c mice containing lesions caused by *L. major* infection. The treatment was carried out for 50 days with different concentrations of curc-AgNPs (20–60 μg/kg). The results indicated a decrease in lesions when compared to the negative control. Furthermore, a significant decline in *L. major* load was observed in 4 weeks.

A different study evaluated the leishmanicidal performance of curc-coated gold nanoparticles (curc-AuNPs) against *L. major* [125]. The NPs showed spherical morphology, particle size of 22 nm, and adequate polydispersity. Curc-AuNPs revealed IC_50_ values against *L. major* promastigotes of 64.79 μg/mL and 29.89 μg/mL in 24 h and 48 h, respectively. Additionally, they inhibited and eliminated amastigotes inside macrophages (IC_50_: 63.29 μg/mL on 24 h and 54.04 μg/mL on 48 h). *In vivo* treatment (BALB/c mice for 50 days) with curc-AuNPs (20–60 μg/kg) reduced lesions caused by *L. major* promastigotes after four weeks. The parasite burden of the paws and lymph nodes of mice infected with *L. major* was also significantly reduced compared to the negative control.

Hence, it is noteworthy that both MNPs had an impact on cutaneous leishmaniasis infections. However, there was no influence of metal (Ag or Au) particles on the leishmanicidal activity of the systems. In these studies, the found activity may be mostly attributed to curc-coated NPs. The same behavior was observed in Miltefosine-AgNPs developed by Kalangi and collaborators. Silver nanoparticles alone (50 μM) did not demonstrate an antileishmanial effect on the promastigote stage of the *Leishmania* parasite. However, when AgNPs were combined with miltefosine (12.5 μM and 25 μM) the leishmanicidal effect of the drug doubled [122]. Therefore, combining leishmanicidal drugs, such as curc, with metallic particles can be an effective strategy against the leishmania parasite.

The formulations described in Section 1.4 are summarized in Table 1, which also describes their physicochemical characteristics and performance *in vitro* and *in vivo*.

**Table 1:** Summary of the curcumin-loaded nanoformulations intended for the treatment of leishmaniasis.

Nanoformulation	Composition	Size/ PdI/ ZP/ EE/ DL	Parasite	*In vitro* and *in vivo* outcomes

SNEDDSs [81]	Captex^®^ 355, Crem EL^®^, Crem RH^®^ 40, Tween^®^ 80, PEG 200, PEG 300, caprylic acid and curc	≈29 nm/ 0.2/ −3.6 to −4.4 mV/ –/ –	*L. tropica*	IC_50_ decreased by 125 times and more than 90 times compared to free curcumin against promastigotes and amastigotes, respectively.
SNEDDSs [48]	Captex^®^ 355, Crem EL^®^, Crem RH^®^ 40, Tween^®^ 80, PEG 200, PEG 300, caprylic acid and curc	30–80 nm/ ≈0.2/ −1.5 to −4.8 mV/ –/ –	*L. tropica*	IC_50_ decreased by more than 86 times and more than 66 times compared to free curc against promastigotes and amastigotes, respectively.
nanoliposomes [89]	phosphatidylcholine, cholesterol, curc	176.5 nm/ ≈0.2/ +35 mV/ 92%/ –	*L. major*	IC_50_ values decreased as the incubation time increased, from 24 h to 72 h.
NLC [96]	glyceryl monostearate, soy phosphatidylcholine, curc	≈312 nm/ ≈0.3/ −38mV/ 88%/ 1.07%	*L. tropica*	NLCs increased the leishmanicidal activity of curc in 1.55 times *in vitro* and 1.28 times *in vivo*.
polymeric NPs [50]	chitosan, mannose, curc	215 nm/ ≈0.3/ +24.73 mV/ 82.12%/ –	*L. donovani*	curc-CNPs and curc-MCNPs showed *in vitro* IC_50_ values (1.5 and 4.5 times, respectively) lower than that of free curcumin. The *in vivo* uptake study showed the endocytosis of NPs by macrophages.
polymeric NPs [105]	chitosan, mannose, curc	215 nm/ ≈0.3/ +24.73 mV/ 82%/ –	*L. donovani*	*In vivo* antileishmanial study showed greater suppression of parasite replication in the spleen by curc-MCNPs compared to curc-CNPs.
polymeric NPs [45]	PLGA, Eudragit^®^ L30D, curc, miltefosine	≈182 nm/ ≈0.2/ −12.7 mV/ ≈93%/ ≈18%	*L. donovani*	Association of curc-E-PLGA-NPs with miltefosine revealed synergism in promastigote and amastigote forms.
metallic NPs [124]	silver nitrate, curc	32 nm/ –/ −19.8 mV/ –/ –	*L. major*	curc-AgNPs (i) prevented the *in vitro* growth of *L. major* promastigotes, and (ii) inhibited their viability; (iii) eliminated amastigotes inside macrophages, and (iv) decreased cutaneous lesions *in vivo*.
metallic NPs [125]	Au, curc	22 nm/ –/ –/ –/ –	*L. major*	curc-AuNPs IC_50_ values decreased as the incubation time increased (24 to 48 h). *In vivo* treatment demonstrated curc-AuNPs reduced cutaneous lesions after four weeks.

Ag: silver; Au: gold; C: chitosan; curc: curcumin; DL: drug load; EE: encapsulation efficiency; M: mannose; MNPs: metallic nanoparticles; NPs; nanoparticles; NLC: nanostructured lipid carriers; SNEDDSs: self-nanoemulsifying drug-delivery systems; PdI: polydispersity Index; PLGA: poly(lactic-*co*-glycolic acid); ZP: zeta potential.

### General discussion and final considerations

This study discussed that curcumin is a polyphenol with diverse biological properties, including a potent leishmanicidal activity. Despite the promising activity, this molecule shows poor water solubility, high metabolism, and fast elimination impair, which results in low systemic bioavailability and poor *in vivo* pharmacological effect. In this context, molecules with similar limitations have been combined with nanotechnology tools to allow their clinical use. In recent years, different nanostructured systems have been explored, such as lipid nanoparticles, metallic nanoparticles, polymeric nanoparticles, nanovesicles, and self-emulsifying nanosystems. These can promote increased solubility, permeability, protection against degradation in biological media, and a controlled release profile. Additionally, nanostructures, especially those smaller than 200 nanometers, are susceptible to uptake by the cells infected with the etiological agent of leishmaniasis. This ability allows an expressive increase in the leishmanicidal activity of curcumin against the parasite.

Despite its versatility, several studies stop at the development of nanostructures containing curc and fail to further assess their activity in the parasites, which slows down the path to a future product. Therefore, only a few studies have evidenced *in vitro* and *in vivo* proof-of-concept that curc nanostructured systems might be promising for the treatment of leishmaniasis. Overall, alternative therapeutic nanostructured systems have been presented. They are nanoliposomes, SNEDDSs, NLCs, polymeric and metallic nanoparticles. These nanostructured systems have different compositions, sizes, charges, and modified surfaces which improves the leishmanicidal activity of curc and other control drugs.

To date, only three *in vivo* studies in mice using nanostructured curc have been carried out. These were all based on polymers and metallic nanostructures. Despite the conducted investigations, there are still gaps regarding a better understanding of the mechanisms of action of curc against leishmaniasis parasites. This review demonstrated that the known aspects related to curc leishmanicidal activity involve the increased production of ROS and intra-amastigote release of Ca^2+^. In this context, we could observe that the studies failed to correlate consecutive intra-macrophage and intra-amastigote cellular uptake kinetics, once it appears to greatly interfere with the proposed biochemical triggers of *Leishmania* spp. cell death.

In addition, the work could also summarize that lipid-based nanostructures are great alternatives. However, the lack of *in vivo* studies on this matter limits their fair comparison to polymeric formulations. Overall, all assessed studies could prove that nanostructures improve curc dispersion in aqueous media (increase in apparent solubility). Altogether, we could also observe a general decrease in IC_50_ when compared to free curc, which was mainly attributed to the cell uptake of these structures. Indeed, studies that functionalized nanostructures with mannose for an increase in macrophage phagocytosis evidenced that functionalized nanoparticles decreased IC_50_ when compared to non-functionalized nanostructures and the free drug. These results take us in the direction of avoiding furtive conditions when developing further nanostructures for leishmaniasis.

Hence, based on the biological potential of curcumin and known safety/tolerability, and based on the existing proof of concept that nanostructured systems are more effective than conventional medicines, reduce the duration of treatment and the frequency of administration, there is an urgent need for industrial innovation towards new treatments for leishmaniasis.

## Conclusion

Given that leishmaniasis is a neglected tropical disease, treatment is still poorly funded. Although the disease is spread in countries with large populations, such as Brazil and India, there are insufficient worldwide investments and not enough priority to prevent this disease from spreading, which makes it deadly to a large part of the population. Due to resistance to many drugs, several research works have focused on optimizing the use of amphotericin B nanoformulations (e.g., AmBisome^®^, Abelcet^®^, and Anforicin B^®^), which is an antifungal and now widely used for leishmaniasis. However, this potent drug is not selective enough to be used in all cases. Present research and future works might still focus on this effective, traditional, and toxic molecule, once there are already different products on the market. However, due to the fast increase in drug resistance, alternatives must be taken into consideration and there is room for new medicines with drugs that are not only effective but less toxic.

This work showed that many nanostructures are being developed and assessed for leishmaniasis on a research level. However, policies and investments that fast-track the development of a nanostructured product from the bench to the market might be key in the future. Although promising, biopharmaceutical limitations still should be regarded and might limit the current studies on nanostructures containing curcumin. Therefore, efforts, time, and resources could be saved by optimizing a single nanostructure for different administration routes, which takes into consideration the biological barriers involved in the treatment of different forms of leishmaniasis (VL, CL, and MCL). This rationale is based on the lack of information observed in the studies regarding skin permeability on lesioned and healed skin, and gastric stability of nanostructured curc *in vivo*.

Also, clinical studies that prove the efficacy and safety of nanostructured curc must be conducted to encourage the transfer of these formulations to the therapeutic scenario. Based on the findings, polymeric nanoparticles reveal themselves to be a step ahead in the game, once more *in vivo* information is available and current medicines based on nanoparticles provide insights for fast-tracking this system from the technological and regulatory point of view.

## Abbreviations

Table 2 lists the in this article used abbreviations and their explanations.

**Table 2:** Explanation of abbreviations.

Abbreviation	Explanation

NTDs	neglected tropical diseases
Curc	curcumin
NLCs	nanostructured lipid carriers
PdI	polydispersity index
SNEDDSs	self-nanoemulsifying drug-delivery systems
NE	nanoemulsions
ME	microemulsions
PNPs	polymeric nanoparticles
APIs	active pharmaceutical ingredients
ED_50_	median effective dose
IC_50_	inhibitory concentration
ZP	zeta potential
NPs	nanoparticles
SI	selectivity index
VL	visceral leishmaniasis
CL	cutaneous leishmaniasis
MCL	mucocutaneous leishmaniasis
DL	drug load
EE	encapsulation efficiency
FDA	Food Drug Administration
